# Exogenous Application of GABA Improves PEG-Induced Drought Tolerance Positively Associated with GABA-Shunt, Polyamines, and Proline Metabolism in White Clover

**DOI:** 10.3389/fphys.2017.01107

**Published:** 2017-12-22

**Authors:** Bin Yong, Huan Xie, Zhou Li, Ya-Ping Li, Yan Zhang, Gang Nie, Xin-Quan Zhang, Xiao Ma, Lin-Kai Huang, Yan-Hong Yan, Yan Peng

**Affiliations:** ^1^Department of Grassland Science, Animal Science and Technology College, Sichuan Agricultural University, Chengdu, China; ^2^Ganzi Prefecture Grassland Station of Sichuan Province, Kangding, China

**Keywords:** γ-aminobutyric acid, polyamines metabolism, proline accumulation, drought stress, white clover

## Abstract

In order to investigate the physiological effects of exogenous γ-aminobutyric acid (GABA) on drought tolerance in white clover (Trifolium repens), GABA shunt, polyamines (PAs), and proline (Pro) metabolism were examined after plants pretreated with or without GABA (8 mM) and then exposed to water or 15% PEG-induced drought stress in growth chamber. In this study, exogenous application of GABA effectively alleviated drought-induced damage in leaves, as reflected by significantly higher relative water content, lower electrolyte leakage, lipid peroxidation, and leaf wilt. Exogenous GABA further promoted drought-induced increases in GABA transaminase and alpha ketone glutarate dehydrogenase activities, but inhibited glutamate decarboxylase activity under both control and drought conditions, resulting in an increase in endogenous glutamate (Glu) and GABA content. Besides, exogenous GABA could well accelerated PAs synthesis and suppressed PAs catabolism, which lead to the extremely enhanced different types of PAs content (free Put and Spd, insoluble bound Spd and Spm, soluble conjugated Spd and Spm, and total Put, Spd and Spm) under drought stress. In addition, exogenous GABA application further activated drought-induced Δ^1^-pyrroline-5-carboxylate synthetase and proline dehydrogenase activities, but suppressed drought-facilitated ornithine -δ-amino transferase activities, leading to a higher Pro accumulation and metabolism in GABA-pretreated plants in the middle and last period of drought. The results suggested that increased endogenous GABA by exogenous GABA treatment could improve drought tolerance of white clover associated with a positive regulation in the GABA-shunt, PAs and Pro metabolism.

## Introduction

Drought stress is one of the most severe limiting factors that negatively affects plant growth and development in perennial forage species around the world (Annicchiarico and Piano, 2004). The scarcity of available water restrains plant productivity due to drought-induced cellular membrane damage, nutritional imbalance, and metabolic disorders. However, plants have evolved a range of physiological, biochemical, and metabolic responses to drought stress including the synthesis or degradation of phytohormone, plant growth regulators (PGRs), osmolytes, and antioxidants defense associated with drought tolerance. Recently, the exogenous application of PGRs is an effective strategy to alleviate drought damage in plant, such as glutamic (Glu), proline (Pro), polyamines (Pas; Yamaguchi et al., 2007; Li et al., 2016b) and γ-aminobutyric acid (GABA; Hu et al., 2015). GABA is a four-carbon non-protein amino acid which could rapidly accumulate when plants suffer from environment stresses (Bown and Shelp, 1997; Medina et al., 2009; Narsai et al., 2011). The biosynthesis of endogenous GABA is mainly involved in two pathways: one is the decarboxylation of Glu from the catalytic action of glutamate decarboxylase (GAD) in the cytosol and the other was from the degradation of PAs through diamine oxidase (CuAO) and polyamine oxidase (PAO) (Bhatnagar et al., 2001).

In recent years, more and more studies have indicated that GABA plays a special role in the regulation of the tolerance to abiotic stress in plants rather than just a metabolite. It had been widely demonstrated that exogenous application of GABA successfully enhanced the tolerance to various abiotic stresses in plants. It was reported that exogenous application of GABA positively regulated the antioxidant defense and photosynthesis in pepper (*Capsicum annuum*) seedlings under low light stress (Li et al., 2017). Also, GABA pre-treating could alleviate the damage induced by chilling stress in tomato (*Lycopersicon esculentum*) seedling, peach (*Primus persica*) fruit as well as wheat (*Triticum aestivum*) seedlings (Shang et al., 2011; Al-Quraan et al., 2013; Malekzadeh et al., 2014). GABA-treated rice (*Oryza sativa*) suffered less heat stress due to the accumulation of increased osmolytes and the up-regulation of antioxidant ability (Nayyar et al., 2013). What's more, exogenous GABA improved the resistance to saline-alkaline in muskmelon (*Cucumis melon*) involved in the protection of photosynthesis apparatus and the alleviation of stress-related photoinhibition (Xiang et al., 2016). For the drought stress, Vijayakumari and Puthur (2015) indicated that GABA application could significantly enhance the drought tolerance through alleviating lipid peroxidation and inhibiting photosynthetic and mitochondrial activity in black pepper (*Piper nigrum*). In addition, application of exogenous GABA also improved drought resistance of creeping bentgrass (*Agrostis stolonifera*) associated with the increased accumulation of amino acids, organic acids, and other osmotic substances related to secondary metabolism (Li et al., 2016a). Moreover, it is proved that the reduction of GABA content closely in relation to a decreased resistance to drought in *Arabidopsis* mutant by the knock-out of glutamate decarboxylase gene (Mekonnen et al., 2016). In conclusion, it is suggested that GABA plays a positive role in physiological modulation when plants suffer from unfavorable environment stresses and the manipulation of GABA levels could be a potential method in enhancing stress resistance in plants.

In plants, GABA shunt exhibits particular roles in the maintenance of carbon/nitrogen balance, regulation of cytosolic pH, scavenging of reactive oxygen species, osmoregulation, and signaling transduction coupling with TCA cycle (Serraj et al., 1998; Bouché and Fromm, 2004; Shi et al., 2010; Krishnan et al., 2013; Mekonnen et al., 2016). In GABA shunt, owing to the loss of GABA transaminase (GABA-T) in *Arabidopsis pop2* mutant lines, *Arabidopsis* generally showed more sensitive to various abiotic stresses (Al-Quraan and Al-Share, 2015). Moreover, the functions of GABA closely linked with PAs and Pro in plants when response to abiotic stress. The catabolism of PAs is an important origin of GABA production and Pro shares the common synthetic precursor glutamic acid with GABA. Hu et al. (2015) demonstrated that exogenous GABA application positively facilitated PA biosynthesis and enhanced endogenous GABA level, which effectively alleviated Ca(NO_3_)_2_ stress damage in muskmelon. Shang et al. (2011) proposed that GABA treatment could well accumulate the content of endogenous GABA and proline, which was useful to protect peach fruit suffering from chilling injury. However, these researches only partly illustrate probable relationships between GABA and other PGRs (Glu, PAs, or Pro), it is still scarce about a comprehensive understanding of the effects of exogenous GABA on GABA shunt, PAs, and Pro metabolic pathways when plants encountered abiotic stress.

White clover (*Trifolium repens*) is an important cool-season forage and turfgrass, which has been widely planted in grassland and landscaping owing to its high crude protein content, nitrogen fixation ability, and excellent ornamental characteristics. However, white clover is very sensitive to limited water supply in production (Annicchiarico and Piano, 2004; Mercer and Watson, 2007). Therefore, it is critical for increasing the drought resistance of white clover through economic and effective strategy. In this study, white clover “Ladino” was pretreated with exogenous GABA in roots before exposed to Polyethylene glycol (PEG, a widely used drought inducer similar to natural water shortage in plants) induced drought stress (Fan and Blake, 1997; Comeau et al., 2010; Kautz et al., 2015). The objectives were (1) to examine whether application of exogenous GABA could alleviate drought damages, (2) to assess the effects of exogenous GABA on GABA shunt, polyamines, and proline metabolic pathway. Such information will be benefited to better understand the physiological adaptability to environmental stress in plants and develop effective cultivation measures to protecting plants from drought stress.

## Materials and methods

### Plant materials culture

The same size and grain plumpness seeds of white clover cv. “Ladino” were selected in this study and all seeds were sterilized with 0.1% HgCl_2_ for 3 min and then rinsed 3 times with distilled water. 0.1 g seeds were sown in each seedlings tray (24 cm length, 20 cm width and 15 cm deep) with pre-sterilized quartz sand and then placed all trays in growth chamber (16 h photoperiod, 75% relative humidity, and 23/19°C day/night) with distilled water for germination 7 days. In subsequent stage, the seedlings were watered with fresh Hoagland's solution (Hoagland and Arnon, 1950) in roots every 2 days.

### GABA pretreatment and drought stress

After 30 days (the second leaves fully expanded), the plants were exposed to four treatments: (1) Control (well watering plants with Hoagland's solution in roots); (2) GABA (pretreating plants for 4 days with 8 mM GABA in roots, watering GABA solution once every 2 days); (3) PEG [watering 15% PEG-6000 (W/V) solution in roots]; (4) GABA+PEG (pretreating the plants with GABA as treatment (2) before PEG treatment). Three hundred and fifty milliliter Hoagland's solution with appointed concentration of GABA or PEG were changed every 2 days in treatment. The GABA concentration was determined by our preliminary experiment. Each treatment had four independent biological replicates, there were 16 pots plants in total. leaf samples were collected at the 0, 10, 17 days of PEG treatment.

### Chemicals treatment *in vitro* condition

To further explore whether a higher GABA concentration plays a positive role in white clover responsing to drought stress, the plant leaves were directly collected and treated *in vitro* for 16 h as follows: (1) Control; (2) 4N (GAD activity inhibitor); (3) AG (CuAO and PAO activity inhibitor); (4) 4N+PEG; (5) AG+PEG; (6) PEG; (7) GABA+PEG. Six independent replicates were applied for each treatment.

### Physiological parameters

#### Relative water content, electrolytic leakage, and malondialdehyde

0.1 g fresh leaves (FW) from each treatment were wrapped in gauze and completely immersed in distilled water, and then put into refrigerator at 4°C for 24 h. Saturated weight (SW) were weighed after the leaves were removed from distilled water and gently dried. Subsequently, leaf samples were put into kraft paper bags in oven, then inactivated at 105°C for 30 min and dried at 75°C for 48 h to determine dry weight (DW). Leaf relative water content(RWC)was determined by using the following formula (Barrs and Weatherley, 1962):

(1) $$\begin{array}{r} \text{RWC}\left(\%\right)=\frac{FW-DW}{SW-DW}\times 100\% \end{array}$$

For the measurement of electrolyte leakage (EL), 0.1 g fresh leaves were put into the tube with deionized water (15 mL). Leaf samples were incubated for 24 h at room temperature and initial conductivity of the solution (C_*initial*_) were determined by using a conductivity meter (YSI Model 32, Yellow Spring, OH). After that, leaf samples were boiled at 100°C for 30 min and cooled to room temperature to measure the final conductivity (C_*final*_). EL was calculated by using the following formula (Blum and Ebercon, 1981):

(2) $$\begin{array}{r} \text{EL}\left(\%\right)=\frac{C_{initial}}{C_{final}}\times 100\% \end{array}$$

Malondialdehyde (MDA) was measured obeying method of Dhindsa et al. (1981). Leaves (0.1 g) was ground on ice bath with 1.5 mL 150 mmol/L phosphate buffer saline (PBS, pH = 7.8). The homogenate was centrifuged at 15,000 g for 40 min at 4°C. 0.5 mL MDA crude enzyme solution (the supernatant after centrifugation) and 1.0 mL mixture [including 20% w/v trichloroacetic acid (TCA) and 0.5% w/v thiobarbituric acid] were incubated at 95°C for 15 min, and then rapid cooled to room temperature on ice bath (shaken to prevent the formation of bubbles). The mixture was centrifuged at 10,000 g for 10 min. The absorbance of the supernatant was determined at 532 and 600 nm. MDA content was expressed as nmol/g DW.

#### Glutamic, GABA content and metabolism enzyme activity

Glu content and Alpha ketone glutarate dehydrogenase (α-KGDH) activity were determined by using Assay Kits (Suzhou Comin Biotechnology Co., Ltd., China) according to the manufacturer's protocol.

The endogenous GABA content was determined by Berthelot reaction with some modifications (Guijin and Bown, 1997). Leaves (0.1 g) were ground with methanol at room temperature. The homogenate was centrifuged at 5,000 g for 15 min and discarded the supernatant (2–3 times). The sediment was re-dissolved in 1.5 mL distilled water. Subsequently, the samples were heated in water bath at 50°C for 2 h, and then centrifuged at 7,000 g for 15 min. One milliliter supernatant was added 0.1 mL 2 mol/L AlCl_3_ and oscillated. The mixture was cooled to room temperature and then centrifuged at 12,000 g for 10 min. The supernatant (0.5 mL) was shaken for 5 min with 0.3 mL KOH and centrifuged at 12,000 g for 5 min. The resulting supernatant was used to measure the content of GABA based on the following procedure: 0.3 mL supernatant was added to the reaction solutions [including 0.5 mL 0.1 mol/L sodium tetraborate (pH = 10.0), 0.4 mL 6% phenol and 0.6 mL 5% sodium hypochlorite]. The mixture was put into a boiling water for 10 min and rapidly placed in ice bath for 5 min. Finally, the solution was shaken with 2 mL 60% ethyl alcohol and measured the absorbance in 645 nm.

GAD and GABA-T activities were determined according to Bartyzel et al. (2003) and Ansari et al. (2005) with some modifications, respectively. 0.1 g leaves were ground to homogenate with 100 mM Tris-HCL (pH 7.5) (including 1 mM EDTA, 1 mM PMSF, and 10% glycerol). The mixture was centrifuged at 12,000 g for 20 min at 4°C. The supernatant was transferred to a new tube and used for determination of GAD and GABA-T activities. Enzyme activity was presented as mmol g^−1^ protein h^−1^.

#### Polyamines content and metabolic enzyme activity

Free, insoluble bound and soluble conjugated PAs (including Put, Spd and Spm) were quantified according to Duan et al. (2008) with some modifications. Leaves (0.1 g) were homogenized with 1 mL perchloriv acid (precool, 5%, v/v) and incubated at 4°C for 1 h. And then the mixture was centrifuged at 12,000 g for 30 min at 4°C. The supernatant was benzoylated as the following process: 500 μl supernatant was blended with 2 mL NaOH (2M) and 10 μl benzoyl chlorides and incubated 37°C for 30 min. Two milliliter saturarted NaCl solution was used to terminate the reaction. In order to extract benzoyl PAs, 2 mL cold diethylether was added into the solution and 1 mL ether phase was evaporated to dryness. The dried product were re-dissolved in 1 mL methanol to determine the content of PAs.

Polyamines biosynthetic enzyme (including ADC, ODC and SAMDC) activities were assayed according to Hu et al. (2012). 0.1 g leaves was ground with 5 mL extract solution (including 50 mmol/L PBS, pH = 6.3, including 5 mmol/L EDTA, 0.1 μmoI/L PMSF, 40 μmoI/L pyridoxal phosphate, 40 μmoI/L insolubility ployvinylpynolidone, 5 mmol/L dithiothreitol and 24 mmol/L Vc) on ice bath. After centrifugation at 12,000 g for 40 min at 4°C, the supernatant was dialyzed against 3 mL of 100 mmol/L PBS (pH 8.0) (including 0.05 mmol/L PLP, 1 mmol/L DTT and 0.1 mmol/L EDTA) for 24 h in darkness at 4°C. 0.3 mL dialyzed enzyme extract solution were mixed with 1.5 mL of reaction system included 1 mL 100 mmol/L Tris-HCl buffer (pH = 7.5, containing 5 mmol/L EDTA, 5 mmol/L DTT and 40 μmoI/L L-Arg for determination of ADC, 40 μmoI/L L-Orn for determination of ODC or 40 μmoI/L determination of S-adenosylmethionine for SAMDC n). Enzyme activity was presented as μmol g^−1^ protein h^−1^.

PAO and CuAO (Polyamines catabolism enzyme) activities were determined according to Su et al. (2005) with some modificatuions. 0.5 g leaves were homogenized with 1.5 mL 100 mM potassium-PBS (pH 6.5) and centrifuged at 10,000 g for 20 min at 4°C. The reaction mixture contained 0.2 mL supernatant, 2.5 mL 100 mM potassium-PBS (pH = 6.5), 0.2 mL 4-aminoantipyrine/N, N-dimethylaniline, 0.1 mL horseradish peroxidase and Spd+Spm (20 mmol/L) for PAO determination or Put (20 mmol/L) for CuAO. The absorbance values were measured at 550 nm for calculating enzyme activity.

#### Proline content and its metabolic enzyme activity

Pro content was measured by the ninhydrin method according to Bates et al. (1973). 0.1 g leaves were extracted in 5 mL 3% sulfosalicylic acid and then heated in boiling water for 20 min. The samples were cooled to room temperature and then 1 mL extract was mixed with 2 mL glacial acetic acid and 3 mL ninhydrin reaction mixture. The reaction solution was heated in boiled water at 100°C for 40 min and then put into ice bath mixing with 2.5 mL toluene. Chromophore-containing toluene was used for final determine the absorbance at 520 nm.

Δ^1^-pyrroline-5-carboxylate synthetase (P5CS) activity was determined according to hydrochloric acid amine colorimetric method (Williams and Frank, 1975). Leaves (0.1 g) were homogenized with 0.5 mol/L Tri-HCl (pH = 7.5), 10 mmo1/L MgC1_2_, 2 mmol/L Benzyl phthalein fluorine, and 2% PVP in ice bath. The homogenate was centrifuged at 20,000 g for 20 min at 4°C. The supernatant were mixed with 3 mL reaction mixture (including 50 mmol/L Tri-HCl, 20 mmol/L MgCl2, 10 mmol/L ATP, 100 mmol/L Hydroxamate-HCl, and 50 mmol/L L-Glu, pH = 7.0) and immediately incubated in 37°C water bath for 15 min. In order to terminate the reaction, 3 mL reaction termination buffer (including 0.5 mmol/L HCl, 5% FeC1_3_ and 12% TCA) were added into the solution. Subsequently the absorbance of the reaction solution was measured at 535 nm.

Ornithine -δ- amino transferase (δ-OAT) activity was analyzed based on Kim et al. (1994). Leaves (0.1 g) were ground with 100 mmol/L PBS (pH = 7.9, including 1 mmol/L EDTA-Na_2_, 10 mmol/L β-mercaptoethanol and 15% glycerinum) and centrifuged at 15,000 g for 15 min. The supernatant were mixed with 50 mmol/L Tris (containing 50 mmol/L L-ornithine, 5 mmol/L α-ketoglutaric acid and 0.05 mmol/L pyridoxal phosphate, pH = 8.0) and put into water bath for 20 min at 37°C. Followed, the mixture with perchloric acid and 2% ninhydrin were heated in boiling water for 5 min and centrifuged at 13,000 g for 30 min. The sediment was redissolved in absolute ethylalcohol. The absorbance of the solution was measured at 510 nm.

Proline dehydrogenase (ProDH) activity was analyzed according to Rena and Splittstoesser (1975) with some modifications. Leaves (0.1 g) were ground with 100 mmol/L PBS (including 1 mmol/L cysteine and 0.1 mmol/L EDTA-Na_2_, pH 8.0) and centrifuged at 15,000 g for 30 min. The supernatant were mixed with enzyme reaction solution (containing 100 mmol/L Na_2_CO_3_-NaHCO_3_ and 20 mmol/L L-Pro, pH = 10.3) and then incubated at 32°C for 5 min. 50 μL nicotinamide adenine dinucleotide (NAD) was added into reaction mixture and immediately monitored with a UV detector at 310 nm.

#### Quantitative real-time PCR (qRT-PCR) analysis

Total RNA of white clover leaves were extracted by using Plant RNA Kit (GBCBIO Technologies Inc., China) according to the specification. An iScriptTM cDNA Synthesis Kit from USA Bio-Rad Laboratories was used for revers-transcripting RNA to first chain of cDNA. The amplification of β*-Actin* was used as an internal control to normalize all data. Primer sequences for β*-Actin, ADC, ODC, CuAO, GAD, PAO*, and *SMADC* were showed in Table 1. The conditions of the PCR protocol for all genes were as follows: 30 s at 95°C and 30 repeats of denaturation at 94°C for 30 s, annealing at 58°C (β*-Actin, ADC, ODC* and *CuAO*) or 63°C (*GAD, PAO* and *SMADC*) for 30 s, 1 min at 72°C, followed 72°C for 5 min.

**Table 1:** All primers used in this experiment.

**Gene**	**Gene no**.	**Primer sequence(5′-3′)**	**Tm (°C)**
*GAD*	KX856932	GCAGCTAGTGGCGGCTTCAT	63
		CGATTCCAGCATAGACAAGACCATA	
*ADC*	KX856933	ATCTGCTGCTACCCTTCGTGG	58
		GCTGTTCAATCCCTAAAGTGCC	
*ODC*	KX856934	AACTTCCAACAGTCAAGCCTTTCT	63
		GGTTGGCATAGATGATTCGGTC	
*SAMDC*	KX856936	GCAGCCAAGATGACCAACAAC	63
		GAAACAGCAGCACCTTCAACAG	
*CuAO*	KX856931	CGAACAAAGCGTTGCGATAGA	58
		GTACTCTTCTCTTCTCCAAACCACC	
*PAO*	KX856935	GAGGTTGCGGGTTCCTGTAGAT	63
		GGCAGCCATTGTTCCAGTAGAGTAT	
*β-Actin*	JF968419	TTACAATGAATTGCGTGTTG	58
		AGAGGACAGCCTGAATGG	

#### Statistical analysis

The data was analyzed by using SPSS 19.0 (IBM, Armonk, New York, USA). The statistical significance among treatments were determined using Fischer's least significance difference (LSD) at the 0.05 probability level.

## Results

### Phenotype, RWC, EL, and MDA content of white clover plants

**Figure 1:**
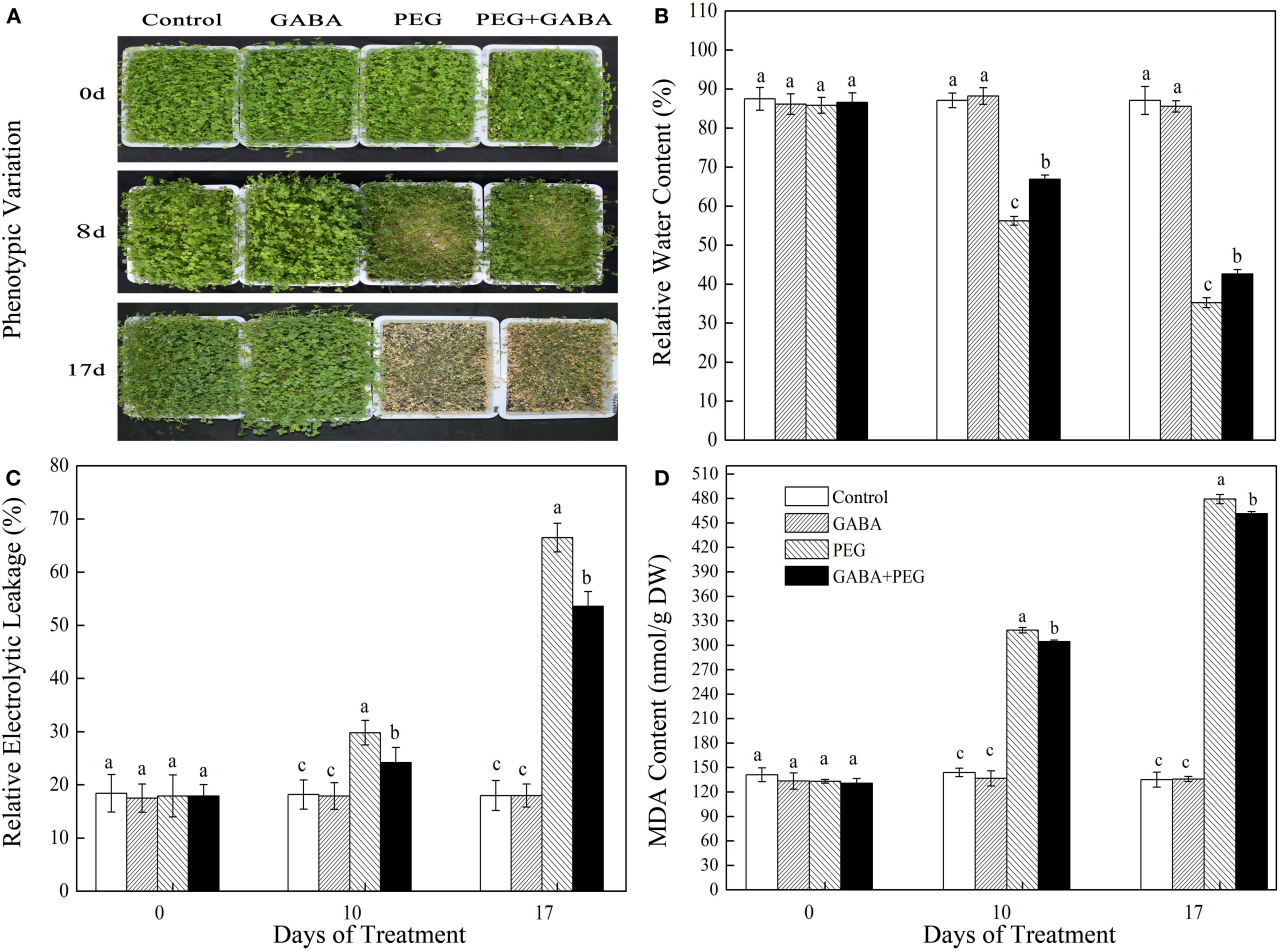
Appearance performance **(A)**, RWC **(B)**, EL **(C)**, and MDA content **(D)** of white clover in different treatments. Vertical columns indicate Mean ± std (*n* = 4). The same letter indicates no significant difference and the different letter indicate significant difference under a particular day of treatment. (*P* < 0.05).

No significant differences were observed in appearance performance, RWC, EL, and MDA content among all treatments under control conditions in white clover (Figure 1). Exogenous application of GABA effectively mitigated drought-induced leaf wilting (Figure 1A). PEG treatment gradually decreased leaf RWC, however, GABA-pretreated plants showed significantly higher RWC compared with PEG treatment alone during PEG-induced drought stress (Figure 1B). Meanwhile, EL and MDA content continually raised with the duration of drought, but GABA application observably inhibited the rising trend (Figures 1C,D).

### Endogenous GABA content and GABA-Shunt metabolism

**Figure 2:**
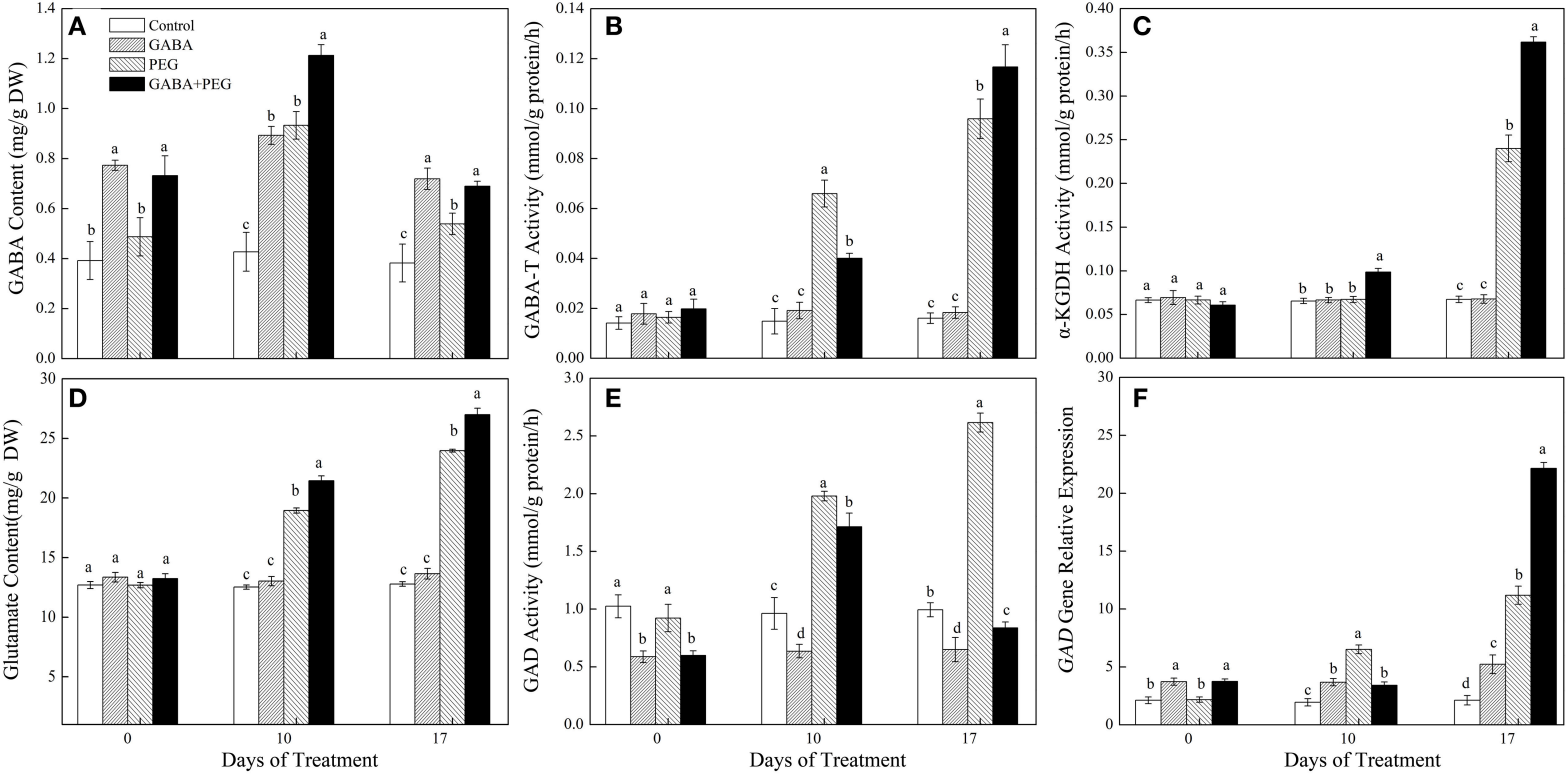
GABA content **(A)**, GABA-T activity **(B)**, α-KGDH activity **(C)**, Glu content **(D)**, GAD activity **(E)**, and GAD gene relative expression **(F)** of white clover in different treatments. Vertical columns indicate Mean ± std (*n* = 4). The same letter indicates no significant difference and the different letter indicate significant difference under a particular day of treatment. (*P* < 0.05).

Exogenous application of GABA greatly enhanced endogenous GABA content in white clover under non-stress condition (Figure 2A). PEG-induced drought stress caused a rapid accumulation of endogenous GABA contents, however, GABA+PEG treatment showed significantly 33.33 and 23.08% higher GABA contents compared with PEG treatment at 10 and 17 days of drought, respectively. The activities of GABA-T and α-KGDH between different treatments had no significant differences under well-watered conditions. Drought induced a gradual increase in GABA-T and α-KGDH activities, but higher GABA-T and α-KGDH activities were observed in GABA pretreatments than in non-GABA pretreatments under drought stress, except an opposite GABA-T activity at 10 days of drought (Figures 2B,C). Compared with PEG treated plants, α-KGDH activities of GABA+PEG treated plants increased by 50.71% at 17 days of drought stress.

Glu content, GAD activity and *GAD* gene expression all gradually increased with prolonged drought stress, and GABA pretreated plants maintained significantly higher Glu content than non-pretreated plants along with a visibly suppression in GAD activity under both control and stressful conditions (Figures 2D–F). GAD activity in GABA+PEG treated plants was 210.71% lower than that in PEG-treated plants at 17 days of drought (Figure 2E). *GAD* gene expression was significantly up-regulated by exogenous GABA under well-watered conditions. A significantly higher of *GAD* gene relative expression levels in GABA+PEG treated plants were detected at 17 days while lower at 10 days when compared to only PEG-treated plants (Figure 2F).

### Endogenous polyamines contents

**Figure 3:**
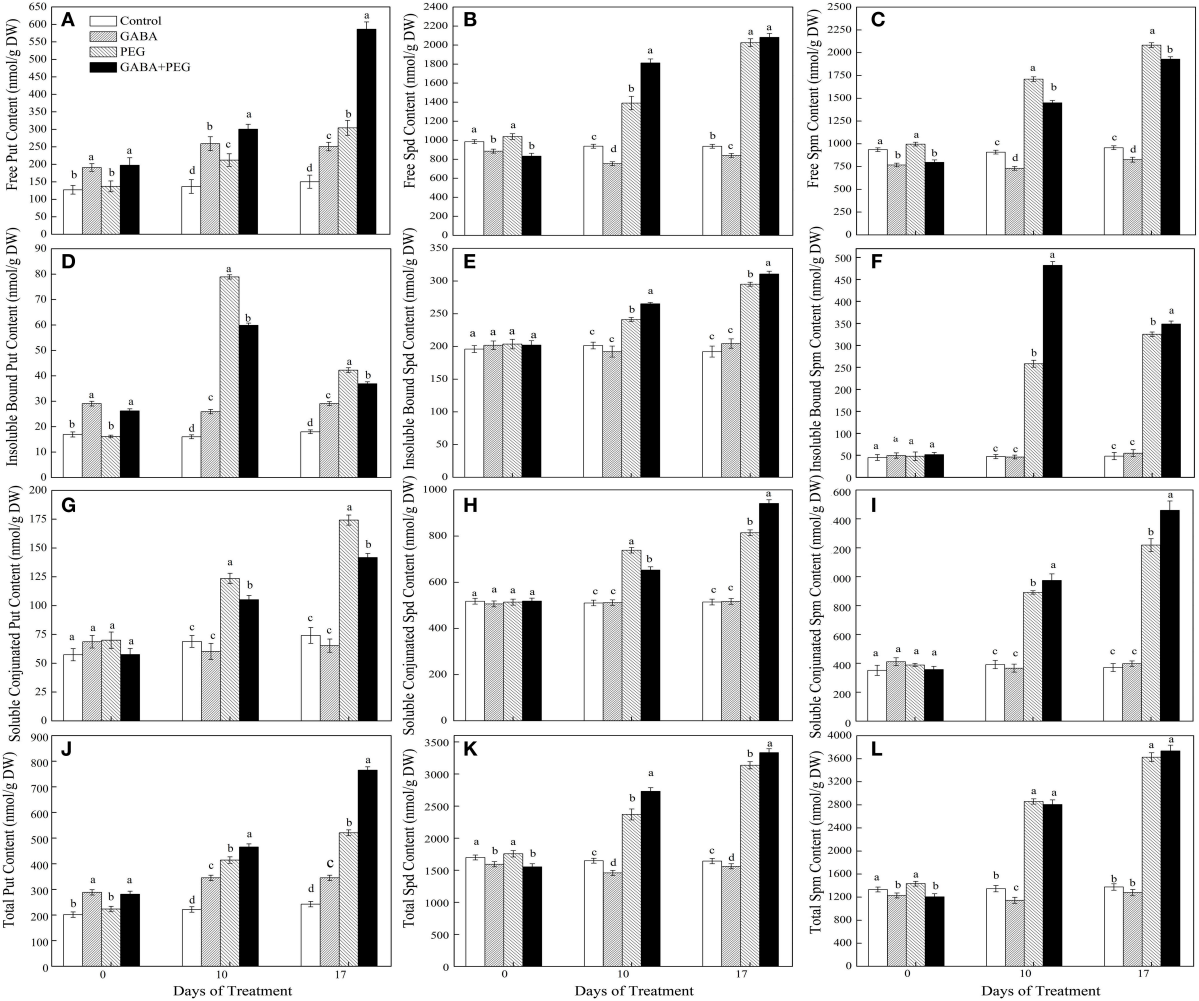
Polyamines content of white clover in different treatments. Free Put content **(A)**, free Spd content **(B)**, free Spm content **(C)**, insoluble bound Put content **(D)**, insoluble bound Spd content **(E)**, insoluble bound Spm content **(F)**, soluble conjugated Put content **(G)**, soluble conjugated Spd content **(H)**, soluble conjugated Spm content **(I)**, total Put content **(J)**, total Spd content **(K)**, and total Spm content **(L)**. Vertical columns indicate Mean ± std (*n* = 4). The same letter indicates no significant difference and the different letter indicate significant difference under a particular day of treatment. (*P* < 0.05).

Under well-watered conditions, treatments with GABA had higher free, insoluble bound and total Put contents than treatments without GABA, accompanied by no significant changes for soluble conjugated Put content in 4 different treatments. Increased free, insoluble bound, soluble conjugated and total Put contents were detected under drought stress. Furthermore, GABA+PEG treated plants showed remarkably higher free and total, but lower bound and conjugated Put contents than PEG treated plants alone (Figures 3A,D,G,J). GABA+PEG treated plants showed 41.65 and 92.69% higher free Put content than PEG treated plants at 10 and 17 days of drought stress, respectively.

Treatments with GABA had lower free and total Spd contents than treatments without GABA under well-watered conditions, meanwhile bound and conjugated Spd contents did not significantly changed. Free, bound, conjugated and total Spd contents obviously increased under PEG-induced drought stress and exogenous application of GABA further enhanced different forms and total Spd contents (Figures 3B,E,H,K). GABA+PEG treated plants showed 30.34 and 10.03% higher free and bound Spd at 10 days, 15.62% higher conjugated Spd at 17 days than PEG-treated plants, respectively.

Exogenous application of GABA significantly decreased free and total Spm contents, and had no significant alteration to bound and conjugated Spm content under well-watered conditions. PEG-induced drought stress greatly promoted free, bound, conjugated and total Spm accumulation in leaves. However, GABA pretreated plants showed significantly lower free Spm content, higher bound and conjugated Spm contents compared with non-treated GABA plants at 10 and 17 days of drought stress. As a result, there was no significant differences in total Spm content between GABA+PEG treated and PEG-treated plants (Figures 3C,F,I,L).

### Polyamines biosynthesis

**Figure 4:**
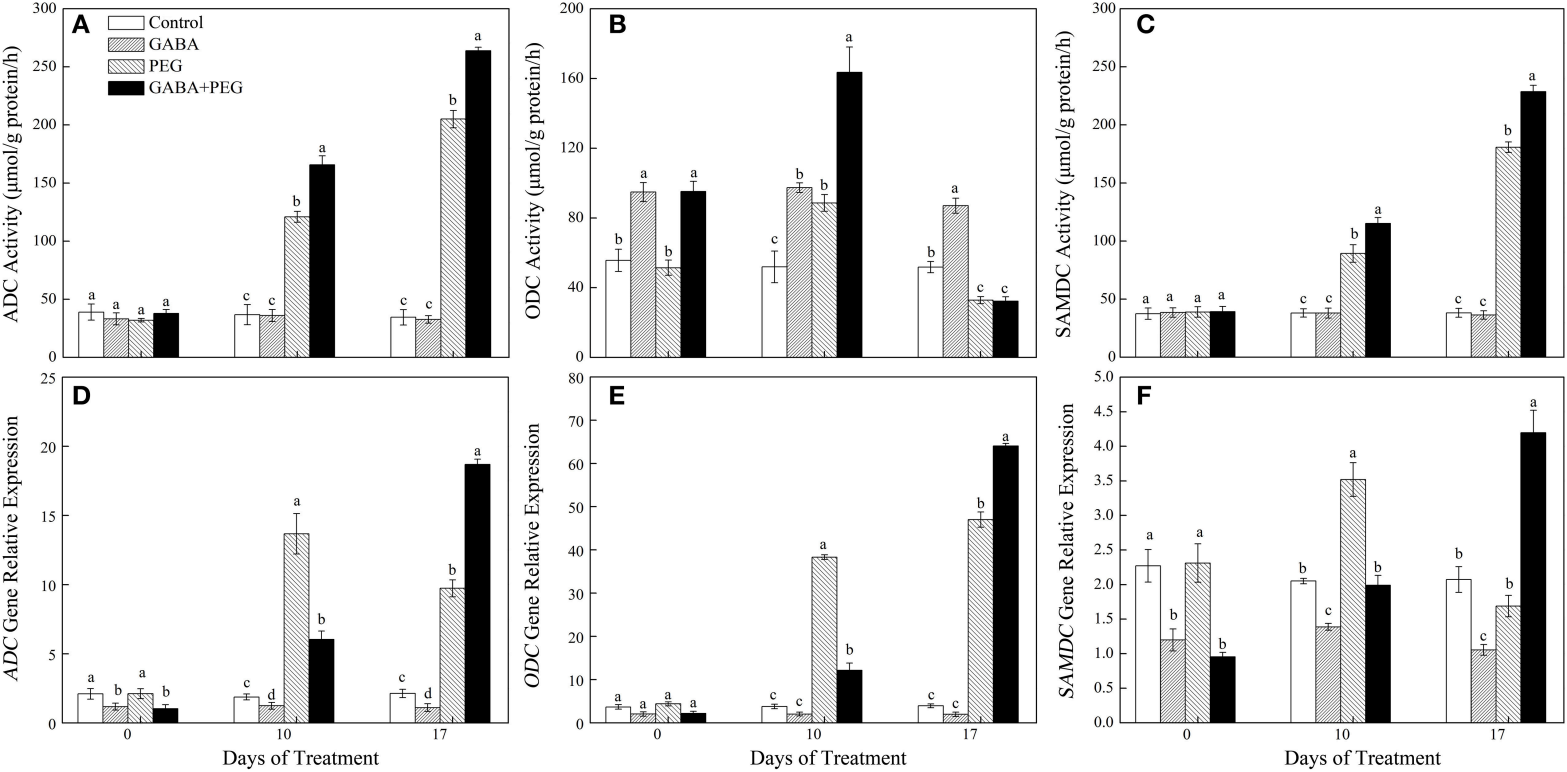
Activities of key enzymes that catalyze polyamine synthesis. ADC **(A)**, ODC **(B)**, SAMDC **(C)** and relative expression of genes ADC **(D)**, ODC **(E)**, and SAMDC **(F)** in white clover leaves under different treatments. Vertical columns indicate Mean ± std (*n* = 4). The same letter indicates no significant difference and the different letter indicate significant difference under a particular day of treatment. (*P* < 0.05).

Under well-watered condition, ADC and SAMDC activities had no significant differences in four treatments, and GABA treatment significantly increased ODC activities (Figures 4A–C). ADC and SAMDC activities were increasingly induced by PEG-induced drought stress, and GABA pretreatment further enhanced this two enzymatic activities during drought stress, but ODC activity only at 10 days of drought (Figures 4A–C). GABA induced a significant decrease in both *ADC* and *SAMDC* gene expression, and had no effect on *ODC* expression under well-watered conditions. Drought stress generated an increase and a decrease in both of *ADC* and *SAMDC* gene expression at 10 and 17 days respectively, and a continuous up-regulation in *ODC* expression (Figures 4D–F). GABA treated plants showed a significantly lower and higher expression in three genes at 10 and 17 days of drought stress, respectively (Figures 4D–F).

### Polyamines catabolism

**Figure 5:**
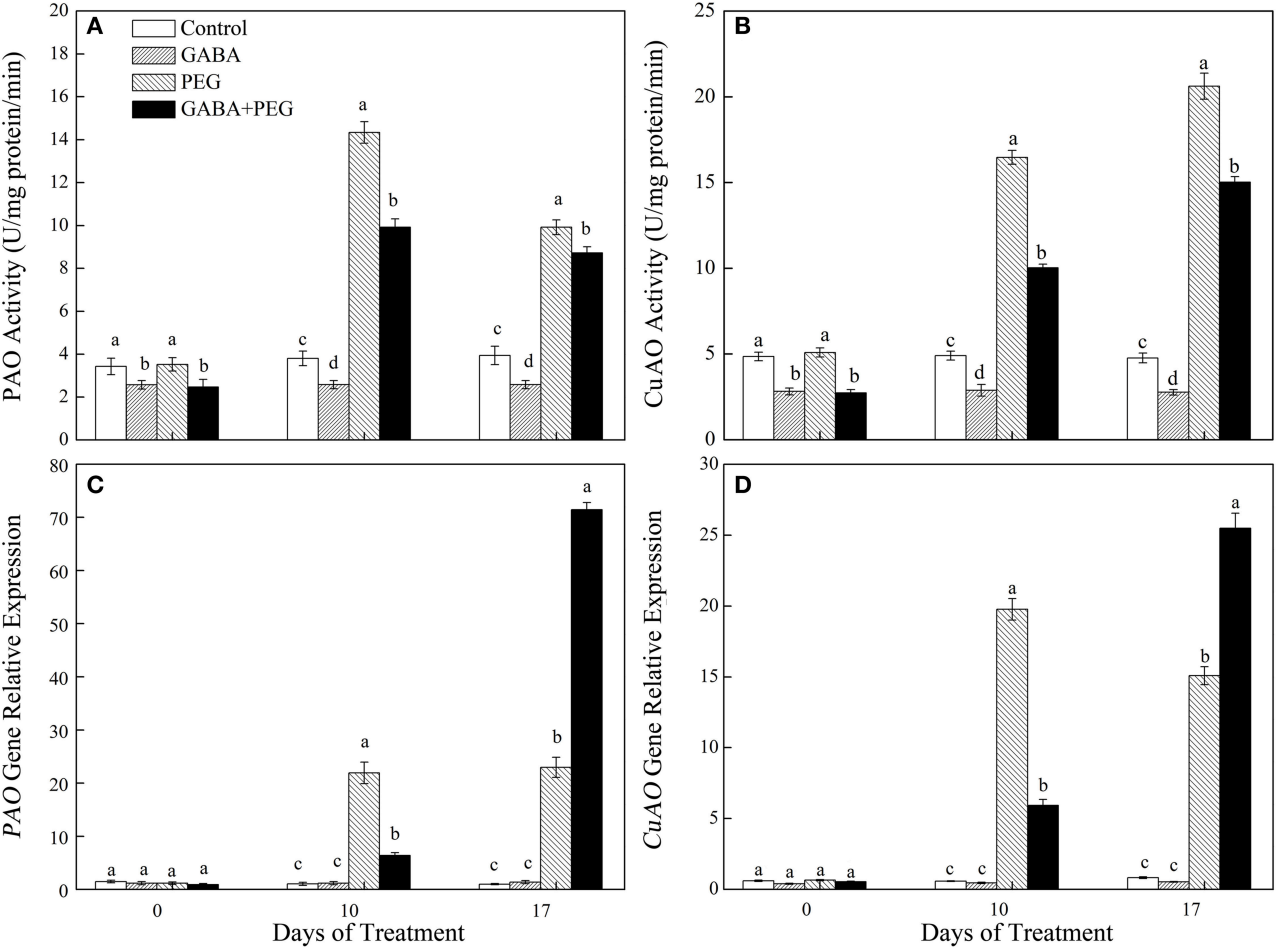
Activities of key enzymes that catalyze polyamine catabolism, PAO **(A)** and CuAO **(B)** and relative expression of genes, PAO **(C)** and CuAO **(D)** of white clover at different treatments. Vertical columns indicate Mean ± std (*n* = 4). The same letter indicates no significant difference and the different letter indicate significant difference under a particular day of treatment. (*P* < 0.05).

Exogenous application of GABA significantly inhibited PAO and CuAO activity under both well-watered and stressful conditions. PEG-induced drought stress up-regulated PAO and CuAO activities and genes expression. Compared with PEG treatment, GABA pretreatment decreased PAO activity by 30.82 and 12.06%, CuAO activity by 39.10 and 27.13% at 10 and 17 days of drought, respectively (Figures 5A,B). GABA had no effect on *PAO and CuAO* genes expression under normal conditions, but GABA pretreated plants showed a greatly lower and higher expression level of this two genes than non-treated plants at10 and 17 days of drought stress, respectively (Figures 5C,D).

### Proline metabolism

**Figure 6:**
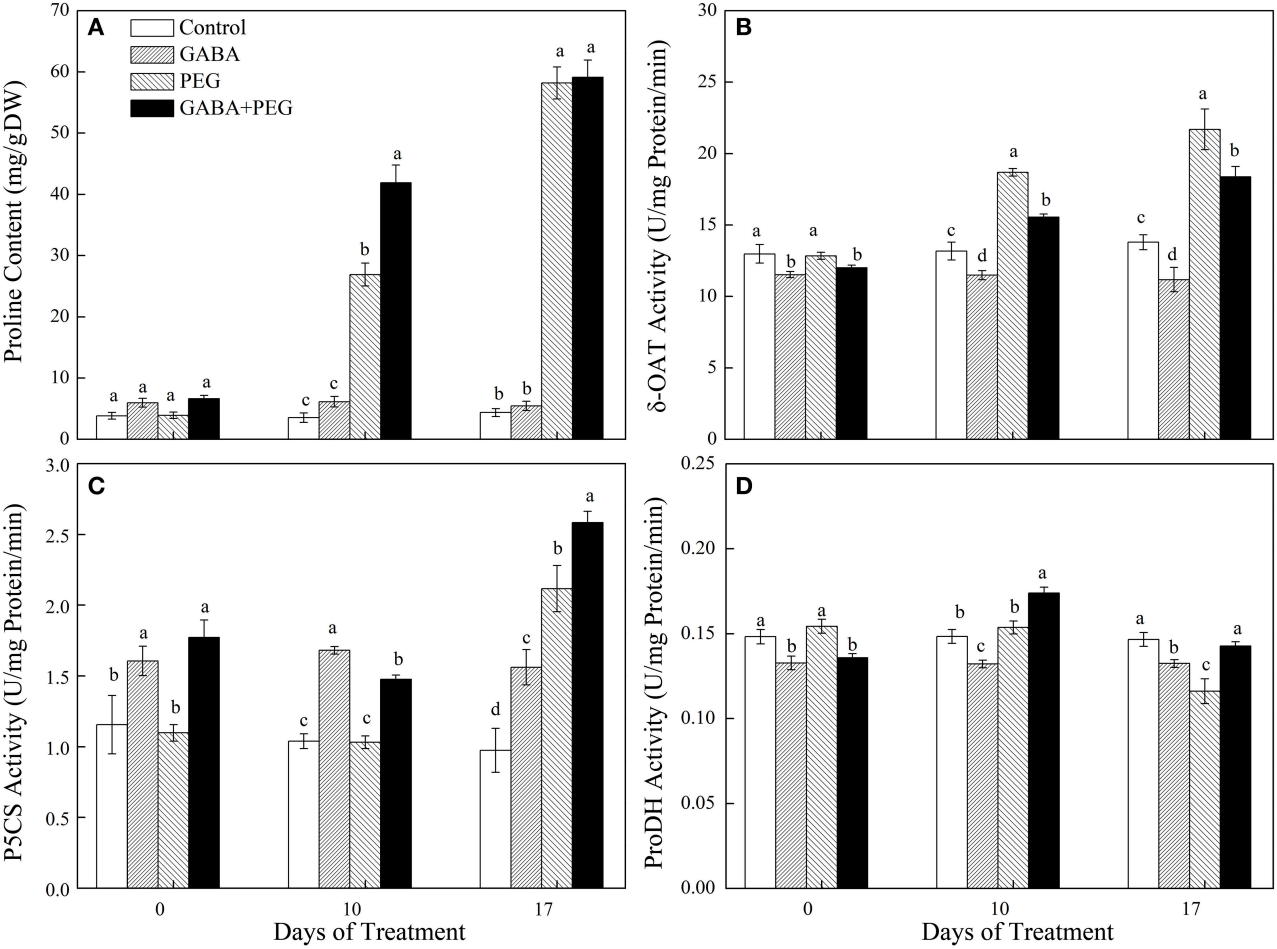
Changes in proline content **(A)** and key enzymes activity of δ-OAT **(B)**, P5CS **(C)**, and ProDH **(D)** of white clover at different treatments. Vertical columns indicate Mean ± std (*n* = 4). The same letter indicates no significant difference and the different letter indicate significant difference under a particular day of treatment. (*P* < 0.05).

Proline contents had no observable difference in all treatments along with significantly reduced δ-OAT and ProDH activity but increased P5CS activity in GABA treatments before PEG-induced drought stress (Figure 6). Drought quickly induced proline accumulation corresponding to boosting δ-OAT and P5CS activity involved in proline synthesis, but restraining ProDH activity responsible for proline degradation (Figures 6A–D). GABA still showed a remarkably restraining effect on the δ-OAT activity, but a stimulative effect on both P5CS and ProDH activity under PEG-induced drought stress, resulting in a 55.76% higher proline content in GABA-pretreated plants compared to non-pretreated plants at 10 days of drought stress (Figures 6A–D).

### Effects of exogenous inhibitor of GABA biosynthesis on physiological variation under drought stress

**Figure 7:**
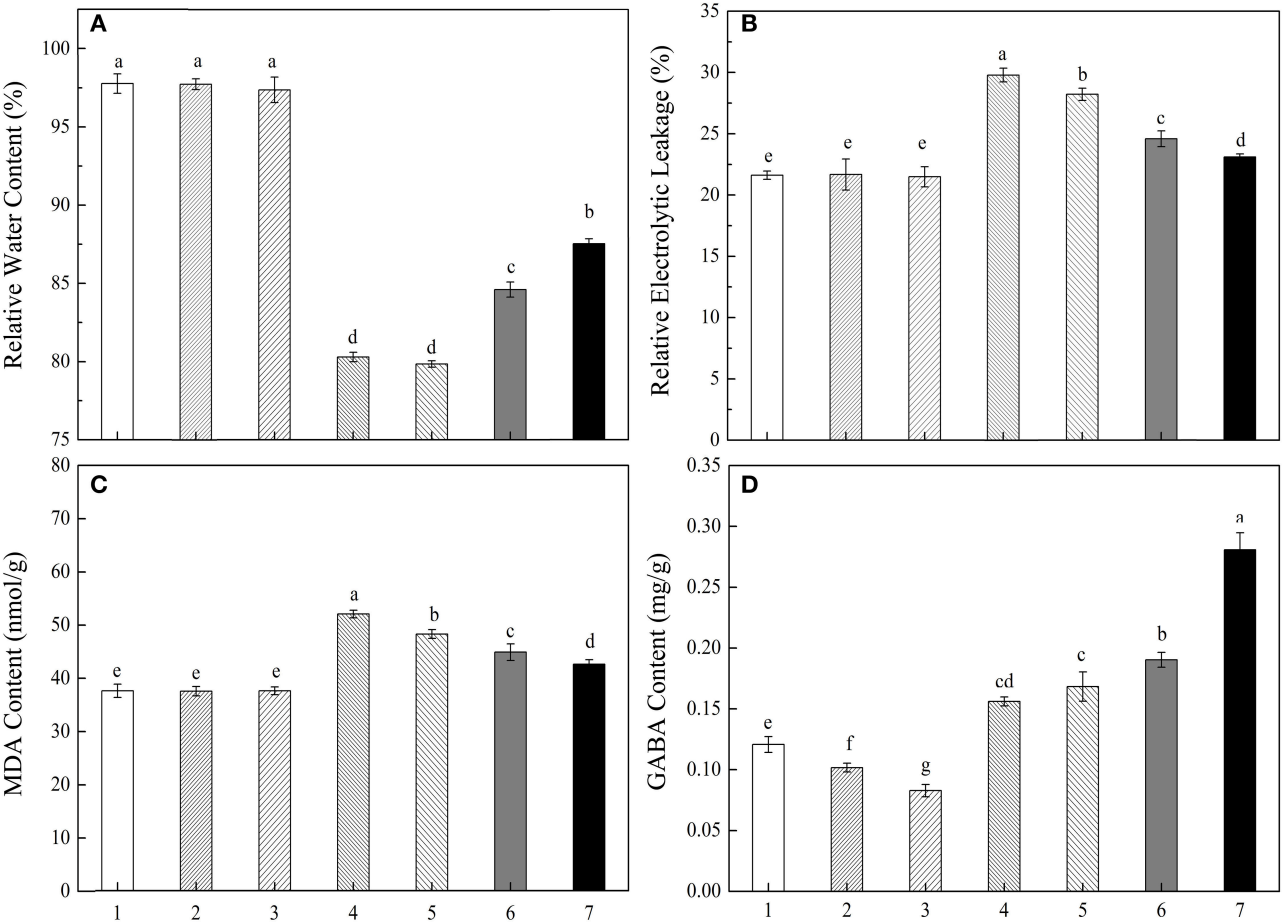
Different treatments on RWC **(A)**, EL **(B)**, MDA **(C)** and endogenous GABA content **(D)** of white clover leaves. The plants were treated with: H_2_O (1);6 mmol/L 4N (2); 1 mmol/L AG (3); 6 mmol/L 4N + 15% PEG (4); 1 mmol/L AG + 15% PEG (5); 15% PEG (6); 0.05 mmol/L GABA + 15% PEG (7). Vertical columns indicate Mean ± std (*n* = 4). The same letter indicates no significant difference and the different letter indicate significant difference between different treatments. (*P* < 0.05).

To further verify the effect of increased endogenous GABA concentration on eliminating drought stress, a pharmacological assay was designed. As shown in Figure 7, both 4N (GAD activity inhibitor) and AG (CuAO and PAO activity inhibitor) could significantly reduce endogenous GABA content of treatments under water and PEG conditions, but the treatment combined GABA with PEG showed significant higher endogenous GABA content than other treatments (Figure 7D). RWC, EL, and MDA content of treatments without PEG had no significant differences. Treatment combined PEG with 4N or AG exhibited significantly lower RWC, higher EL and MDA content than only PEG treatment. Meanwhile, treatment combined PEG with GABA showed the opposite pattern compared with only PEG treatment (Figures 7A–C).

## Discussion

In plants, GABA was firstly discovered in potato (*Solanum tuberosum*) tubers in 1949 (Steward et al., 1949). Subsequently, more and more literatures reported that endogenous GABA obviously accumulated when plant suffering from various stresses (Alan and Frank, 2000). Meanwhile, transporters and receptors of GABA were found successively. It was reported that drought could increase the activities of ProTs and AAP3 (GABA transporters) which regulate GABA entering into plant cells across cell membrane (Rentsch, 1998; Ramesh et al., 2017). In addition, GABA could regulate the release of Ca^2+^ from the intracellular Ca^2+^ store by bounding to its receptors (GLRs and ALMTs) and then modulate the activity of GAD (Cholewa et al., 1997; Malhó, 1999; Lancien and Roberts, 2006). Although these findings, the metabolic effects of GABA on drought tolerance is still not fully understood in plants. Therefore, this study was designed to explain how the GABA concentration affects the metabolic pathway associated with GABA synthesis and catabolism in white clover response to drought stress.

### GABA accumulation could effectively relieve drought damage of plants

Increased EL and MDA accumulation were considered as the indicators of membrane damage and lipid peroxidation induced by excessive ROS generation during stress. White clovers gradually showed the symptom of wilting accompanied by the decrease of RWC as well as the increases of EL and MDA content during PEG-induced water stress. However, pretreatment with GABA effectively alleviate drought-induced leaf wilting and improve membrane stability (decreases in EL and MDA content) under drought stress. The positive effects of exogenous supply of GABA on mitigating drought damage through the maintenance of membrane stability were also found in perennial ryegrass (*Lolium perenne*) and black pepper (Krishnan et al., 2013; Vijayakumari and Puthur, 2015). These results suggested that GABA played an important role in protecting plants from oxidative stress. The pharmacological assay further confirmed the beneficial effects of elevated endogenous GABA level on the alleviation of drought-induced damage in white clover.

### GABA shunt and stress tolerance in plants

Three enzymes involved in GABA shunt are detected in this study. GAD is a cytosolic acidification-activated enzyme which catalyzes irreversible decarboxylation of glutamate to generate GABA. The other two steps of GABA shunt proceed in mitochondria associated with TCA cycle. GABA is reversibly converted into SSA by GABA-T and then SSA is oxidized to produce succinate by SSADH linking with TCA cycle. The potential functions of GABA shunt involved in the balance of C/N metabolism and the maintenance of integrality of TCA cycle have been well documented (Fait et al., 2008). In our experiment, application of exogenous GABA significantly increased the endogenous GABA concentration, which was similar to the findings of Hu et al. (2015) and Wang et al. (2014). Our pharmacological assay also proved that both Glu and PAs pathway contributed to the increase of endogenous GABA concentration. In addition, a higher endogenous GABA concentration and a lower GAD activity were also found under both control and drought conditions, implicating a feedback inhibition between GABA and drought-induced GAD activity. A similar result has been found in hypoxin-stressed melon roots (Wang et al., 2014). A direct suppression of GAD activity induced by exogenous GABA might make a greater contribution to the increase in Glu content due to the decrease in Glu consumption for GABA synthesis.

Interestingly, the pathway of GABA catabolism was further accelerated by exogenous GABA application due to higher activities of GABA-T and a-KGDH. Particularly, GABA-T catalyzes GABA to glutamate or alanine at the first step of GABA degradation. In addition, a-ketoglutarate dehydrogenase (a-KGDH) which catalyzes a-ketoglutarate into succinate in TCA cycle was regarded as a major enzyme for linking GABA shunt and TCA cycle (Bouché and Fromm, 2004). Therefore, the improvement of GABA-T and a-KGDH activities indicated higher GABA catabolism and subsequent TCA metabolic process, resulting in a rapid accumulation of Glu. Besides, lower GAD activity and greater GABA catabolism accounted for the increased Glu content in GABA treatments, which was also found in *Brasska napus* and rice when suffered from drought stress (Good and Zaplachinski, 1994; Do et al., 2013).

The physiological function of GABA-T and a-KGDH has been well described under normal condition, but was scarcely revealed in stress. Hu et al. (2015) found that GABA-T activity in muskmelon was increased by exogenous GABA application under Ca(NO_3_)_2_ stress. It was reported that GABA-T was more functional importance in preventing ROS species accumulation, linking N and C metabolism under stressful conditions (Renault et al., 2010; Al-Quraan and Al-Share, 2015). In our current study, it is observed that endogenous GABA concentration in plants presented an obvious decrease after reaching the peak in the middle stage of drought. This expenditure on GABA was just coordinated with dramatically increased GABA-T and a-KGDH activities in the last stage of drought, suggesting that GABA might flow into the TCA cycle (Fait et al., 2005; Renault et al., 2010). Taking these results into account, it could be speculated that a decreased GABA synthesis and an increased GABA catabolism induced by exogenous GABA application could play a positive role in the alleviation of drought stress damage to white clover through supplying enough Glu and keeping the fluency of GABA shunt and TCA metabolism under drought stress.

### Alteration of endogenous GABA concentration affects polyamines metabolism under drought stress

PAs ubiquitously distribute in all eukaryotic cells and mainly include Put, Spd, and Spm which consist of free, conjugated and bounded status in higher plants (Hu et al., 2012). PAs synthesis originates from three pathways and involves three major enzymes: ADC, ODC, and SMADC. ODC or ADC catalyzes the decarboxylation of ornithine or arginine, which is committed step of Put synthesis. The conversion of S-adenosylmethionine into decarboxylated S-adenosylmethionine could be catalyzed by SAMDC, which donates aminopropyl groups to Put to produce Spd or Spm via Spd synthase or Spm synthase, respectively. In addition, PAs catabolism is catalyzed by CuAO and PAO (Mattoo et al., 2010; Rangan et al., 2014). PAs accumulation has been observed in different plants under environmental stresses and the key roles of PAs have been well confirmed in plants (Sequera-Mutiozabal et al., 2016). A direct link of GABA and PAs is that PAs catabolism produces GABA via CuAO or PAO catalysis. The investigation of Yang et al. (2013) revealed that PAs degradation offered approximately 30% of GABA content in fava beans (*vicia fara*) under hypoxic stress. In our current study, drought improved three PAs synthesis enzymes (ADC, ODC and SMADC) activities and their gene expression levels as well as two PAs catabolism enzymes (CuAO and PAO) and their gene expression, resulting in increases in three types of PAs. The increased PAs biosynthesis and catabolism was also detected in other plants under stressful conditions (Wang et al., 2014; Hu et al., 2015). Furthermore, GABA enhanced PAs synthesis, but had an inhibitory effect on PAs catabolism, resulting in a higher total PAs content in GABA-treated plants under drought condition. This result implied that the increased endogenous GABA had a negative feedback effect on PAs degradation, which was consistent with findings of Wang et al. (2014).

The overexpression of PA biosynthesis enzymes (*ADC, ODC*, and *SAMDC*) and exogenous application of PA all support that higher PAs content provide better drought resistance in different plant species including rice, fava bean and white clover (Capell et al., 2004; Yang et al., 2013; Li et al., 2016b). In our present study, drought stress induced the increase of three forms of PAs (free, conjugated, and bound), which was consistent with the research of Liu et al. (2006) in maize. GABA further increased total contents of Put, Spd, and Spm in addition to free Put, three forms of Spd, conjugated and bound forms of Spm content, but simultaneously decreased the content of conjugated and bound Put as well as free Spm content. These changes suggested that GABA application did not increase the total content of different PA types but induced a conversions of conjugated and bound Put into free Put, and free Spm into conjugated and bound Spm.

Previous studies reported the correlation between Put level and stress tolerance. For examples, transgenic *Arabidopsis* plants over-producing Put conferred drought tolerance (Alcázar et al., 2010). Liu et al. (2004) reported that drought-sensitive wheat cultivar accumulated higher free Put level and the conversion of free Put to conjugated Put could contribute to the drought tolerance. Most of researches in favor of that the conversion of free Put to free Spd and Spm could be helpful in increasing stress tolerance (Duan et al., 2008; Alcázar et al., 2010). In our study, we detected an obvious conversion of conjugated and bound Put to free Put, but not free Put to free Spd and Spm in GABA-treated plants under drought stress. This implied that a higher free Put could play a positive role in coping with drought stress because of its active physiological function.

With respect to the conversion of free Spm to conjugate and bound form Spm, the similar result was also detected in other plants in response to stressful conditions (Wang et al., 2014). Due to the physiological functions of conjugated and bound PAs involved in stabilizing protein structure and keeping membrane integrity and free forms of PAs as a temporary resource, this conversion could also be physiological important (Roussos and Pontikis, 2007; Trovato et al., 2008). Taking these results together, it could be presumed that both GABA-induced PAs accumulation and conversions of different forms of PAs implicate in drought tolerance in white clover in our current study.

### Increased endogenous GABA concentration could regulate proline accumulation in white clover in response to drought stress

It has been well known that plant tissues extensively accumulate free Pro during abiotic stress. Recent studies provided the evidence that multiple functions of Pro were closely involved in its metabolism pathway. Pro is synthesized via two pathways from either Glu or ornithine (Orn). Among which, Glu is reversibly catalyzed by P5CS involved in the synthesis of Pro and δ-OAT catalyzes the conversion of Orn to Pro (Parida et al., 2008). Earlier Vitro assays showed that Pro could scavenge hydroxyl radicals (·OH), which is considered as a most reactive type of ROS species and responsible for serious oxidative damage to cell in stressed plants (Smirnoff and Cumbes, 1989). The work of Hong et al. (2000) further intensified the protective role of Pro in transgenic tobacco (*Nicotiana tabacum*) under oxidative stress. More recently, Signorelli et al. (2014) presented a non-enzymatic way to form GABA via Pro reaction with ·OH under oxidative stress, suggesting a direct connection between Pro and GABA. In our experiment, exogenous GABA kept Pro content as a low level as control under normal conditions resulting from increased P5CS activities and decreased δ-OAT and ProDH activity which is involved in the degradation of Pro. Under drought stress, GABA further activated drought-induced P5CS activities and compensated drought-induced the decrease in ProDH activity, but had a depression effect on drought-facilitated δ-OAT activities, resulting in a higher and similar Pro accumulation in GABA-treated plants compared with non-GABA-treated plants in the middle and last period of drought, respectively. These findings indicated that GABA promoted Pro accumulation and maintained Pro homeostasis under drought stress. However, more and more recent researches approved that a balance between Pro synthesis and catabolism instead of an excessive pro accumulation, played a vital role in plants tolerance against drought stress in addition to traditional functions as compatible solute, ROS scavenger, and energy supply (Sharma et al., 2011; Bhaskara et al., 2015). Therefore, we proposed the positive effects of exogenous GABA on alleviating the oxidative stress associate with Pro accumulation and homeostasis in white clover under drought condition.

## Conclusion

**Figure 8:**
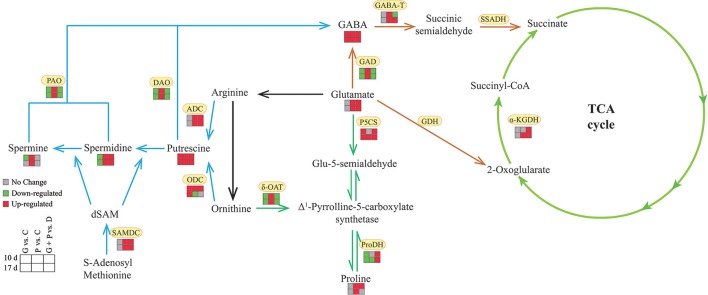
Possible model for exogenous application of GABA improved drought tolerance in white clover associated with GABA-shunt, polyamines and proline metabolism. Blue lines, polyamines metabolism; orange lines, GABA shunt; dark green lines, proline metabolism; light green lines, TCA cycle.

In this study, we detected the effect of exogenous GABA application on drought tolerance and metabolic changes of GABA, Pro, PAs synthesis and degradation in white clover under drought conditions (Figure 8). The increased endogenous GABA concentration induced by exogenous application of GABA effectively reduced drought damage in white clovers. A higher endogenous GABA concentration suppressed GABA synthesis, but enhanced GABA catabolism, resulting in an increase of glutamate content. In addition, exogenous application of GABA also improved PAs synthesis, but inhibited PAs catabolism. As a result, the total content of three types of PAs were further increased, along with a conversion of conjugated and bound Put into free Put as well as free Spm into conjugated and bound forms. The increased activities of P5CS and ProDH and the inhibited δ-OAT activities could account for a higher proline accumulation in the middle period of drought. Based on these findings, we proposed that enhanced concentrations of Glu, PAs and Pro, the conversion of Put and Spm forms, and Pro homeostasis induced by exogenous GABA could play positive roles in alleviating drought-induced damage in white clover.

## Author contributions

YP, BY, and HX conceived the project and designed the experiments; BY, HX, and Y-PL performed the experiments; BY, HX, ZL, and YZ analyzed the data; HX, ZL, YZ, and GN finalized the manuscript; X-QZ, XM, L-KH, Y-HY discussed the results and reviewed the manuscript.

### Conflict of interest statement

The authors declare that the research was conducted in the absence of any commercial or financial relationships that could be construed as a potential conflict of interest.
